# Improvement of rheological and sensory properties of *Lactobacillus helveticus* fermented milk by prebiotics

**DOI:** 10.1016/j.fochx.2024.101679

**Published:** 2024-07-20

**Authors:** Xuelu Chi, Qingyu Yang, Yufang Su, Jian Zhang, Baoguo Sun, Nasi Ai

**Affiliations:** a Key Laboratory of Geriatric Nutrition and Health (Beijing Technology and Business University), Ministry of Education, Beijing 100048, China; b National Center of Technology Innovation for Dairy, Hohhot 010110, China

**Keywords:** *Lactobacillus helveticus*, Fermented milk, Flavour profiles, Inulin, Galactooligosaccharides, Rheological properties

## Abstract

The fermentation characteristics and aroma-producing properties of Lactobacilli could influence the flavour quality of fermented milk, an important influencing factor of consumers' preference. In this study, fermented milk was prepared using *Lactobacillus helveticus* and the dynamic changes in the sensory quality of fermented milk throught fermentation were to assess the dynamic changes in sensory quality of fermented milks throughout the fermentation process, including rheological properties and flavour profiles. Styrene, linalool, octanoic acid, and 1-nonanol were considered as the key flavour components during fermentation. The quality of the fermented milk tends to be stabilized after 24-h, showing the minimal off-flavour at 48-h and optimal fermented aroma at 72-h. Three prebiotics (inulin, Galactooligosaccharides and inulin mixed with Galactooligosaccharides) were added to Lactobacillus spiralis fermented milk separately, and the results showed that inulin mixed with Galactooligosaccharides was the most effective group in improving the organoleptic quality of the fermented milk. Overall, the experimental results provide deeper insights into the release and retention of aroma compounds during fermentation and scientific reference for broadening the application of prebiotics and flavour-producing Lactobacilli in fermented milk processing.

## Highlights

• Four key flavour substances were generated during fermentation by *Lactobacillus helveticus*.
• Styrene was identified to be closely related to the fermented aroma.
• Nonanoic acid and acetic acid significantly affected the off-flavour.
• Prebiotics can effectively improve the sensory quality of fermented milk.

## Graphical abstract

**Figure:**
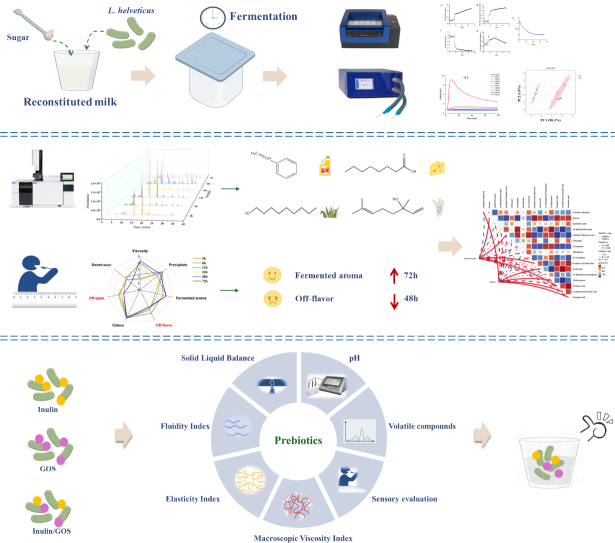

## 1 Introduction

Recently, the dairy industry has been manufacturing products with different nutritional values, textures, and flavours using different starters and/or probiotic strains to achieve marketing requirements (Bai et al., 2020; Liang et al., 2024; Yildiz & Ozcan, 2018). In a previous study, the bioactive components were converted into other metabolites using the starter culture during fermentation, which significantly improved the sensory properties of fermented milk (Liang et al., 2024). Among the lactic acid bacteria (LAB), *Lactobacillus helveticus (L. helveticus)* has been widely applied in the production of dairy products with improved functionality and health benefits, including the potential in terms of mental health (Chelladhurai, Ayyash, Turner, & Kamal-Eldin, 2023), gut well-ness (Liang et al., 2024), cognitive (Dahiya & Nigam, 2022), flavour, texture, and shelf life of fermented milk products (Chelladhurai et al., 2023; Ge et al., 2022; Sıçramaz, Güven, Can, Ayar, & Gül, 2022). Additionally, *L. helveticus* has been reported to enhance the antioxidant, antimicrobial, and lactose tolerance effects of fermented milk products (Chelladhurai et al., 2023; Sıçramaz et al., 2022).

Prebiotics are substrates that are selectively utilized by host microorganisms conferring health benefits (Gibson et al., 2017). Prebiotics could not only control the texture and rheology of fermented milk but also create a symbiotic fermented dairy product to meet the multidimensional needs of consumers (Yu et al., 2021). As a naturally occurring polysaccharide, inulin is mostly found in the roots and rhizomes of various plants (Akram et al., 2024). Besides the prebiotic and antioxidant activities, inulin has been reported to serve as a fat replacer and texture modifier in low-fat dietary fermented milk to enhance the textural and sensory properties, that forms a smooth, creamy texture that provides a silky mouthfeel while delivering a feeling of satiety. The addition of carbohydrate-based substances could induce dynamic changes in the sensory perception, viscoelastic, and lubrication properties (Bayarri, Chuliá, & Costell, 2010; Kieserling, Vu, Drusch, & Schalow, 2019). Meanwhile, the addition of long-chain inulin exhibits a rheological behaviour closer to that of full-fat yogurt and can improve the creaminess of low-fat yogurt (Oliveira, Perego, Oliveira, & Converti, 2011). Conversely, inulin substitution with milk fat affects various preference characteristics, such as appearance, colour, and texture, but does not improve the odour and flavour of yogurt (Glibowski & Rybak, 2015). What's more, inulin cannot be digested by the body and can provide a fat-like texture without increasing energy intake (Damin, Minowa, AlcÂNtara, & Oliveira, 2008).

Galactooligosaccharides (GOS) are among the most extensively evaluated prebiotics consisting of one glucose molecule bound to a chain of one to eight galactose molecules (Elizabeth Tymczyszyn, Gerbino, Illanes, & Gómez-Zavaglia, 2011; Raza et al., 2021). GOS are often added to infant formulas as a beneficial element and used in dairy applications, such as milk yoghurts, buttermilk (Čurda, Rudolfová, Štětina, & Dryák, 2006), and dairy-based drinks ascribe to its excellent solubility. Generally, GOS cannot be destroyed by bacteria, so they remain unmetabolized until reaching the large intestine and have excellent acid stability, making them a suitable ingredient for fermented milk (Sangwan, Tomar, Singh, Singh, & Ali, 2011). Carbon sources exert a significant effect on the growth and energy release of fermenting bacteria, but the dynamics of flavour with the addition of prebiotics are not clear (Cao et al., 2019; Raza et al., 2021). Numerous studies have demonstrated the ability of L. *helveticus* to improve the flavour and mouthfeel of fermented milk. Although these studies were not systematic and in-depth, they were used as a reference to suggest the possibility of flavour improvement (Cao et al., 2019).

Therefore, the present study aimed to explore prebiotic fermented milk from a flavour perspective. Studies on the micro-rheological properties of food containing prebiotics and symbiotics have shown that prebiotics plays a definitive role in the perceived texture properties and flavour release of certain products. In this context, exploring the effect of prebiotics on the flavour characteristics during fermentation has high significance in producing new fermentation foods with improved quality. The present study aimed to: (1) Conduct qualitative and quantitative analyses of Volatile Organic Compounds (VOC) in fermented milk and explore the contribution as the key flavour active compounds of fermented milk. (2) Identify the stable time in fermented milk according to the fermentation characteristics and sensory attributes; (3) Explore the promotion significance of prebiotics on fermentation properties and flavour release through multivariate data analysis method. This study provides novel insights into the mechanism of flavour formation and guidance of quality regulation in fermented milk, offering directions for sensory improvement of fermented milk at the molecular level.

## 2 Materials and methods

### 2.1 Materials

*L. helveticus* B-1 was isolated from Tibetan kefir grain and preserved at the Dairy laboratory at Beijing Technology and Business University, Beijing, China. Whole milk powder was purchased from Fonterra Co-operative Group Ltd. (Auckland, New Zealand). The Man-Rogosa and Sharp (MRS) medium was procured from AOBOX Co., Ltd. (Beijing, China). The prebiotics, including inulin and galacto-oligosaccharides were procured from LuAn Biotechnology Limited Co., Ltd. (Shanghai, China). Sodium chloride (AR) was procured from Sinopharm Chemical Reagent Co., Ltd. (Beijing, China). Butanol and n-alkanes (C7 – C40) were purchased from J&K Scientific Limited Co. Ltd. (Beijing, China).

### 2.2 Sample preparation

The MRS medium was sterilized at 121 °C for 15 min. L. *helveticus*, stored at −80 °C, was cultured in MRS medium at 37 °C for 18 h and stored at 4 °C after two successive subcultures.

The whole milk powder was reconstituted to 8% (*w*/*v*) total solids in the water at 25 °C, and then these mixtures were mechanically agitated using laboratory disperser to obtain uniform distribution of sucrose (5%) in the whole milk powder/water blend. Later, the samples were pasteurized at 65 °C for 30 min, cooled to the incubation temperature of 37 °C, and L. *helveticus* (at least 10^6^ CFU/mL) was added to the milk at 2% (*V*/V) to produce fermented milk. Sucrose was added as a sweetener and milk was fermented at 37 °C. Subsequently, samples were collected at 3 h, 6 h, 12 h, 24 h, 48 h and 72 h and stirred for 10 min before further analysis.

Different prebiotics (3% (w/v)) were added to the reconstituted milk and the other treatments remained unchanged from the control. The addition of inulin was denoted as “inulin”, the addition of GOS was denoted as “GOS”, and the addition of a mixture of inulin and prebiotics (1:1, V: V) was denoted as “inulin/GOS”.

### 2.3 Rheological properties

The microrheological parameters including elasticity index (EI), melt viscosity index (MVI), fluidity index (FI) and solid liquid balance (SLB) of fermented milk were determined by the method of Bai et al. (Bai et al., 2020). The fermented milk (20 mL) was transferred to a micro rheometer (LAB 6 MASTER, Formulation Inc., Toulouse, France) to monitor the microrheological parameters during gel formation at 37 °C.

The pH alteration during milk fermentation was determined via iCinac dairy fermentation monitor (AMS-Alliance, France).

### 2.4 Flavour characteristics of fermented milk

#### 2.4.1 Extraction of flavour compounds

The 8 mL of the fermented milk sample was transferred to a 20 mL headspace vial, mixed with 1 g of NaCl and 10 μL of butanol (internal standard, 2.75 mg/L), and the vial was sealed. After being incubation in headspace vials at 60 °C for 15 min, the VOCs were extracted using a DVB/CAR/PDMS fiber (50/30 μm, 1 cm, Supelco, Bellefonte, PA, USA) for 20 min. Then the fiber was inserted into a GC injector and desorption at 250 °C for 5 min.

#### 2.4.2 GC–MS analysis

The volatiles of fermented milk were analysed by 7890–5975 gas chromatograph-mass spectrometry (Agilent, USA) equipped with a polyethylene glycol capillary column DB-WAX (30 m × 0.25 mm, 0.25 μm film thickness, Agilent, USA).

Flavour analyses were performed according to the method by Chi, Zhang, Zheng, Wang, and Liu (2024) with slight modifications. The column was initially heated to 40 °C and maintained for 3 min, then ramped up to 140 °C at a rate of 5 °C/min (held for 2 min), followed by an increase of 10 °C/min to 240 °C with a 1-min hold. The mass spectrometer was operated in full scan mode and data acquisition was set up in the *m*/*z* range of 35–500.

#### 2.4.3 Identification and quantitation of the volatile compounds

The volatiles were semi-quantified based on the linear relationship between the peak area and concentration of the internal standard (butanol, 2.75 mg/L) and volatile compounds, following the literature with slight modifications (Chi et al., 2024; Huang et al., 2024). Tentative identifications were based on matching mass spectra of unknowns with those in the NIST 2014 database with a threshold of an ion matching score >80%.

#### 2.4.4 Odour activity value (OAV)

OAV analyses were performed to gain the contribution of each component to the overall flavour. The formula is (Xu et al., 2024):

$$\mathrm{OAV}=\frac{C}{T}$$

where *C* and *T* represent the concentration and order threshold of the target compound.

### 2.5 Electronic nose

Overall odour characteristics were obtained via PEN3 *E*-nose (Win Muster Airsense Analytics Inc., Schwerin, Germany) equipped with a metal oxide semiconductor sensor array containing 10 sensors. When the VOCs pass through the instrument, the “odour fingerprint” is detected by the sensors. Before injecting, the fermented milk samples were placed in a vial and incubated it for 300 s at 40 °C under constant stirring. The data acquisition time was 120 s, and the flushing time was 300 s (Chi et al., 2022).

### 2.6 Sensory evaluation

The quantitative descriptive analysis (QDA) was conducted in the sensory evaluation laboratory. All the fermented milk samples were performed by six well trained panelists from the School of Food and Health at Beijing Technology and Business University. The odour intensity values of each sample were rated using a 0 to 5 score method (Supplementary Table 1).

All participants acknowledged an informed consent statement to participate in the study. Ethical approval for the involvement of human subjects in this study was granted by Beijing Technology and Business University Research Ethics Committee under Reference number 44, 2024.

### 2.7 Statistical analysis

All experiments were conducted in triplicate, and the results were presented as mean ± standard error of the mean. The correlation heat map analysis was performed at https://www.omicstudio.cn/tool. The principal components analysis (PCA) was performed by SIMCA14.1(Sartorius Stedim Data Analytics AB, Umea, Sweden). Radar chart and response curve were prepared using Origin Pro 2022 (Origin Lab, Northampton, MA, USA), and the graphs were generated using GraphPad Prism v9.0.1 (GraphPad Software, San Diego, California, USA).

## 3 Results and discussion

### 3.1 Fermentation characteristics

**Fig. 1:**
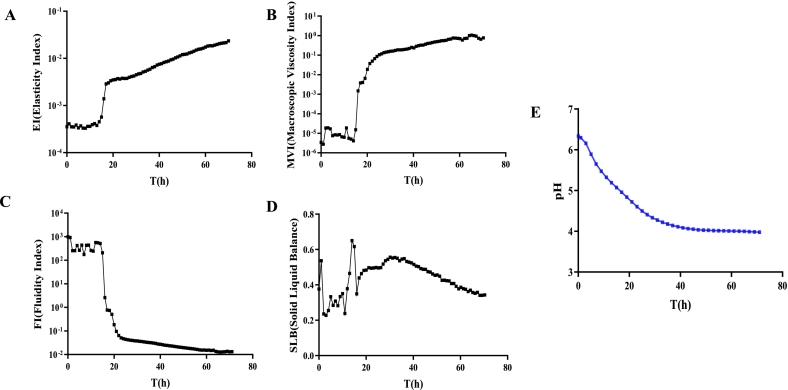
Microrheological properties and pH of fermented milk during fermentation progress. Elasticity index (A); Macroscopic Viscosity Index (B); Fluidity Index (C); Solid-Liquid Balance (D); and pH (E).

As the basic theory of fluid stability, rheological properties play a vital role in yogurt processing and quality control (Wu et al., 2023). The fermentation properties were measured by a micro-rheometer, allowing the physical stability of the fermented milk to be forecasted. The dynamic change in the microrheological parameters, such as EI, FI, MVI, and SLB, were monitored during fermentation (Fig. 1A-D).

At the initial stage of fermentation, insignificant changes were observed in the EI values, which increased rapidly after 15 h and then continued to increase with the fermentation time, indicating that the interaction between emulsion droplets was enhanced. EI value is related to structure formation. During fermentation, the lactose of reconstituted milk was utilized by microorganisms and converted into lactic acid, which leads to acidified milk gels. Tiny droplets in the milk rapidly aggregate to form a flexible structure in the form of a gel network. Therefore, the high EI and MVI value might possess that the fermented milk system has better stability (Tan, Wang, Chen, Niu, & Yu, 2016).

**Fig. 2:**
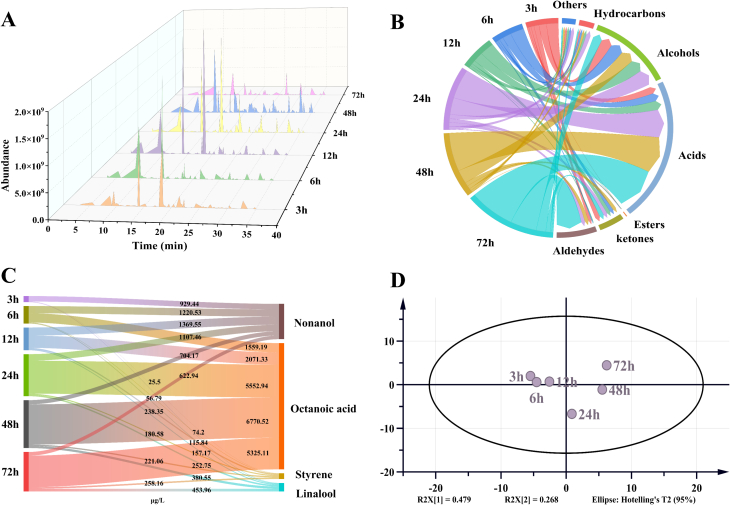
Total ion current chromatogram of VOCs from samples during fermentation (A); The categories and concentration of the volatiles (B); The concentration of key aroma-active compounds (C); PCA score scatter plot (D).

The MVI values represent the fluidity of the droplets in the fermented milk system. Like the dynamic change of EI values, the low viscosity of fermented milk was observed at the initial stage, which appeared to be relatively unstable (Fig. 2B). Additionally, a positive influence of droplet elasticity and viscosity was observed on emulsion stability. After 16 h of fermentation, MVI values were increased rapidly and stabilized at a high level, indicated a high viscosity stage of the samples and a stable gel structure.

FI represents the movement speed of the fermented milk's microscopic particles (Wu et al., 2023; K. Xu, Guo, Du, & Zhang, 2019). Generally, the mobility of a droplet in a system is negatively correlated with MVI. The FI values decreased rapidly from 125 to 0.08 after 15 h of fermentation, then the decrease rate became slower after 21 h, and finally stabilized at a lower level with increased fermentation time. With the viscosity increased, the fluidity of the fermented milk was correspondingly decreased.

The SLB value peaked at 0.76 after 15 h of fermentation but was in an unstable stage during the pre-fermentation period. Then, it reached 0.55 after 32 h of fermentation and continued to decrease and eventually stabilized below 0.5. According to the microrheological tests, the fermented milk showed intrinsic solid properties at about 40 h of fermentation (Tisserand, Fleury, Brunel, Bru, & Meunier, 2012).

The pH can significantly affect the sensory characteristics and microbial activity of the final product (Xu et al., 2019). At the initial stage of fermentation (about 1 h), the pH value was >6.0 and decreased slowly with the extension of fermentation time, then decreased rapidly to around 4.5, and finally stabilized approximately at 4.0 after 40 h (Fig. 1E). During fermentation, *L. Helvetius* induced acid production, increased the number of microorganisms, and accumulated the organic acid, while the low acidity environment correspondingly promoted fermentation. The pH change in fermented milk is closely related to the growth and metabolism of strain during fermentation. As the pH continues to decrease, the growth and metabolism of the LAB are gradually inhibited, eventually reaching equilibrium. The dynamic changes in the sweet-sour attribute of fermented milk are mainly due to the acid-producing capacity of the strain.

According to the results of rheological parameters and pH, the fermented milk reached stability after 30 h of fermentation. Therefore, the flavour release analysis of fermented milk should focus on the period from 30 h to 72 h.

### 3.2 The performance of flavour in fermented milk

#### 3.2.1 Compounds identified by GC–MS

A total of 57 volatile components were identified during fermentation using HS-SPME/GC–MS, including alcohols, aldehydes, ketones, alkenes, esters, hydrocarbons, and others.

Aldehydes are the common flavour substances in fermented milk. With the extension of fermentation time, many aldehydes detected in the early stage disappeared, and then some new aldehydes are produced. The early aldehydes might have come from the sample or heat treatment, or from the metabolism in the rapid proliferation stage of lactic acid bacteria during early fermentation. Aldehydes could easily be reduced to alcohols or oxidized to acids (Tian, Xiong, Yu, Chen, & Lou, 2023). The concentration of benzaldehyde in fermented milk decreased continuously with the extension of fermentation time. Benzaldehyde is slowly converted to other substances, such as phenol and benzoic acid, as a precursor in yogurt, thereby decreasing the concentrations. Nonanal had a fruity flavour and was detected at all time points, and its concentration remained essentially stable. *Trans*-2-Octenal, mainly originated from the lipoxygenase metabolism of unsaturated fatty acids, had an orange peel flavour, and its concentration gradually decreased and eventually disappeared with the progression of fermentation. Disclosing the phenylalanine in fermented milk undergoes Strecker degradation to form large amounts of benzaldehyde during the pre-fermentation phase. As the pH values decreased during fermentation progress, lactic acid bacteria continue to proliferate.

Ketones are mainly derived from the oxidative and thermal degradation of unsaturated fatty acids and the metabolism of amino acids by lactic acid bacteria (Tian et al., 2019; Tian, Yu, Yu, & Chen, 2020). A total of 9 ketones were detected during fermentation. Among them, diacetyl, mainly derived from citric acid metabolism, has a creamy aroma and has been repeatedly detected as a key flavour substance in yogurt. In this study, diacetyl was detected in samples after 72 h of fermentation. Ethyl diphosgene, a reduced form of diacetyl, has a buttery aroma, which is much lower than that of diacetyl. As the fermentation proceeded, the concentration of diacetyl tended to increase, and ethyl diphosgene was detected earlier than diacetyl, which indicated that ethyl diphosgene was produced first, followed by diacetyl, indicating that ethyl diphosgene was produced first, followed by diacetyl, and ethyl diphosgene was accumulated at a higher concentration during fermentation. Additionally, geranyl acetone and 3,5-octadien-2-one appeared only in the pre-fermentation period and decreased in concentration with the extension of fermentation time. 2-heptanone, 2-nonanone, 2-undecanone and 2-tridecanone derived from raw milk, the Maillard reaction during heat treatment, or metabolism of milk fats to fatty acids by LAB, are commonly found in yogurt and can be converted to various methyl ketones through β-oxidation or direct decarboxylation.

Esters are produced through the esterification reaction between alcohols and carboxylic acids or amino acids. Generally, most esters present floral, fruity, and wine aroma that can reduce the bitterness and other odours produced by fatty acids and amines (Güler, 2007; Tian et al., 2023). Herein, esters were found in lower concentrations and less diverse in all samples, but they have important contributions to flavour profile due to their fruity and cheese aroma. During fermentation by L. *helveticus*, the type and content of esters in samples varied considerably. Isobutyl butyrate was detected only at the initial stage (3 h) of fermentation but disappeared with the extension of fermentation time. Isobutyl butyrate was detected in cheese, although the author considered that esters were not produced by the organism but formed during the extraction of the culture distillate or concentration of the extract (Centeno, Tomillo, Fernández-García, Gaya, & Nuñez, 2002). Ethyl octanoate and butyl decanoate were detected at the end stage of fermentation with high concentrations, which have been reported to contribute sweetness as odour active volatiles in fermented brown milk (Wang et al., 2024).

During fermentation, the concentration and types of acid compounds increase, intensifying the sour flavour. However, excessive acids could bring a particularly pungent odour and rancidity to fermented milk. A total of 7 acids were detected during fermentation, including acetic acid (stimulating), capric acid (pungent), caprylic acid (fruity), nonanoic acid (fatty), capric acid (sour odour), 9-decenoic acid, and lauric acid. Acids are important flavour substances in dairy products and are most abundant throughout the fermentation process, and L. *helveticus* has been reported to have a high acid-producing capacity. Caproic, caprylic, and capric acids were the most abundant of all acids, while the other acids were less abundant.

Alcohols are mainly derived from fatty acid reduction, methyl ketone oxidation, amino acid degradation, and sugar metabolism. Alcohols were the most diverse substances detected in all samples and had a significant impact on fermented milk flavour despite their high thresholds. About 9 alcohols were detected in all samples, with isopentenyl, n-hexanol, linalool, geraniol, and α-pinitol showing an increasing trend in concentration.

Hydrocarbons, mainly derived from fat, sugar and amino acid metabolism, were found at low levels in the samples and their thresholds were high, contributing less to the flavour of the yogurt (Tian et al., 2023). The hydrocarbons detected in all samples were dipentene and tetradecane, of which dipentene, also known as limonene, is mainly derived from the metabolism of citric acid and has lemon and orange aroma.

Despite the low concentrations, pyrazines, furans, ethers, pyrroles, and sulfur-containing compounds possess special aromas and contribute prominently to the overall flavour of yogurt. Among them, sulfur-containing compounds are mainly derived from amino acid metabolism, especially the degradation of methionine, which has a cabbage-like flavour. Pyrazine compounds mainly produce barbecue-like aroma. 2-methylfuran has a green bean and butter-like odour, theophylline has a sweet fragrance odour, and toluene has a paint-like odour. Among these compounds, toluene, 2,6-dimethylpyrazine, 2-pentylfuran, 2-methylpyrazine, 2,5-dimethylpyrazine, and butylated hydroxytoluene were detected in all samples as flavourists and their concentrations did not change significantly throughout the fermentation process.

Fig. 2A displays the total ion current chromatograms, with additional details on VOCs are available in Table 1. The dynamic changes in the composition and content of volatiles reflected the growth and metabolism state of L. *helveticus* during fermentation progress (Fig. 2B). The highest levels of aldehydes, ketones and acids were detected in the fermented milk at 72 h. The results of the composition of compounds that not only new substances are produced during fermentation, but the content of some compounds is cumulative and reaches its highest peak after 72 h of fermentation. The total amount of alcohols, hydrocarbons and other compounds increased during fermentation, but after 24 h of fermentation, the total content begins decreased and reached its lowest value at 72 h. At the beginning of fermentation, the total content of aldehydes and ketones decreased but then increased after 6 h of fermentation.

**Table 1:** The results of flavour substances during fermentation of L. *helveticus.*

No.	compounds	CAS	RI	RI_ref_	concentration (μg/L)					
					3 h	6 h	12 h	24 h	48 h	72 h
	Aldehydes (7)									
1	Acetaldehyde	75–07-0	694	713	–	–	–	1782.52 ± 105.67	1519.5 ± 122.57	14,242.28 ± 1724.07
2	Octanal	124–13-0	1279	1286	158.23 ± 13.56	–	–	–	–	–
3	Nonanal	124–19-6	1384	1395	497.97 ± 15.08ab	374.59 ± 50.63bcd	275.37 ± 13.26d	412.63 ± 68.48abc	541.50 ± 88.74a	311.13 ± 74.00 cd
4	(E)-2-Octenal	2548-87-0	1416	1434	23.08 ± 2.80	62.19 ± 18.71	53.93 ± 5.34	–	–	–
5	Benzaldehyde	100–52-7	1508	1520	944.90 ± 75.99	–	–	–	123.68 ± 18.80	121.03 ± 9.35
6	Citral	5392-40-5	1715	1718	48.89 ± 3.81	22.81 ± 1.15	–	–	–	–
7	Tetradecanal	124–25-4	1908	1931	55.60 ± 16.75	–	–	–	–	–
	Total aldehydes				1728.67	459.59	329.30	2195.15	2184.68	14,674.44

	Ketones (13)									
8	Butane-2,3-dione	431–03-8	957	965	–	–	–	–	–	382.40 ± 80.13
9	2-Heptanone	110–43-0	1178	1184	564.68 ± 27.77	179.56 ± 43.42	–	–	494.72 ± 65.76	335.89 ± 53.58
10	Acetyl methyl carbinol	513–86-0	1275	1271	–	–	–	134.12 ± 31.84	234.02 ± 65.60	628.33 ± 100.11
11	6-Methyl-5-hepten-2-one	110–93-0	1328	1323	74.31 ± 19.91	55.82 ± 0.97	67.83 ± 12.38	–	–	–
12	2-Nonanone	821–55-6	1378	1387	851.97 ± 112.87a	703.38 ± 76.07ab	707.56 ± 109.64ab	609.14 ± 23.94bc	479.26 ± 99.12c	462.34 ± 43.36c
13	3-Octen-2-one	18,402–82-9	1396	1411	10.59 ± 2.13	–	–	–	–	–
14	Menthone	10,458–14-7	1479	NF	60.70 ± 0.15	56.21 ± 12.35	33.22 ± 3.00	–	–	–
15	3,5-Octandien-2-one	30,086–02-3	1558	1516	36.38 ± 1.42	–	40.84 ± 7.34	–	–	–
16	2-Undecanone	112–12-9	1586	1599	890.86 ± 112.62bc	843.60 ± 79.77c	1047.27 ± 55.40ab	1139.48 ± 41.23a	615.12 ± 187.79d	577.71 ± 78.30d
17	1,6-Dihydrocarvone	20,379–99-1	1613	NF	–	12.53 ± 1.30	–	14.81 ± 4.89	–	–
18	2-Tridecanone	593–08-8	1796	1814	190.12 ± 23.43a	185.59 ± 31.53a	220.38 ± 15.87a	194.07 ± 46.42a	47.21 ± 28.21b	53.57 ± 10.24b
19	(E)-6,10-Dimethyl-5,9-undecadien-2-one	3796–70-1	1844	1856	26.46 ± 3.76	27.82 ± 2.53	–	–	–	–
20	2-Pentadecanone	2345-28-0	2009	2023	63.66 ± 10.71ab	56.92 ± 33.13ab	30.67 ± 2.20b	125.30 ± 24.20a	53.93 ± 81.39ab	11.79 ± 0.12b
	Total ketones				2769.73	2121.43	2147.77	2216.92	1924.26	2452.03

	Esters (4)									
21	Isobutyl butyrate	539–90-2	1158	1160	8.48 ± 2.72	–	–	–	–	–
22	Ethyl caprylate	106–32–1	1425	1420	–	–	38.98 ± 5.61	60.56 ± 2.4	60.74 ± 17.13	44.44 ± 6.63
23	Amyl pelargonate	61,531–45-1	1823	NF	–	–	–	230.81 ± 6.86	–	–
24	γ-Decalactone	705–86-2	2187	2203	29.98 ± 6.79	–	9.93 ± 2.37	–	16.74 ± 6.24	10.09 ± 0.82
	Total esters				38.46		48.91	291.37	77.48	54.53

	Acids (8)									
25	Acetic acid	64–19-7	1466	1433	–	48.42 ± 0.00	37.9 ± 10.22	763.58 ± 96.60	4642.57 ± 398.55	5558.47 ± 408.61
26	Caproic acid	142–62–1	1851	1827	–	–	4.25 ± 0.86	2953.57 ± 251.56	6742.87 ± 1134.67	9915.47 ± 1418.45
27	Octanoic acid	124–07-2	2078	2050	1154.60 ± 178.89c	1559.19 ± 276.47c	2071.33 ± 292.08c	5552.94 ± 607.39b	6770.52 ± 1544.76a	5325.11 ± 322.26b
28	Cyclohexanecarboxylic acid	98–89-5	2118	2084	–	–	–	–	167.13 ± 25.09	148.98 ± 8.05
29	Nonanoic acid	112–05-0	2196	2144	–	–	–	229.32 ± 3.82	165.33 ± 20.50	193.72 ± 30.14
30	Decanoic acid	334–48-5	2302	2279	2557.06 ± 372.33bc	2278.03 ± 366.63c	2237.99 ± 381.92c	5131.99 ± 921.25a	2365.37 ± 347.15c	3417.87 ± 683.97b
31	9-Decenoic acid	14,436–32-9	2371	2335	–	393.33 ± 75.37	413.37 ± 8.96	742.58 ± 32.67	702.02 ± 86.16	383.29 ± 6.37
32	Lauric acid	143–07-7	2526	2508	512.10 ± 60.38c	618.55 ± 120.84bc	857.51 ± 26.81b	1884.23 ± 245.88a	817.57 ± 167.98b	715.97 ± 177.3bc
	Total acids				4223.76	4897.52	5622.35	17,258.21	22,373.38	25,658.88

	Alcohols (12)									
33	Isopentenol	123–51-3	1204	1185	119.04 ± 8.07a	130.71 ± 49.38a	133.14 ± 14.10a	140.63 ± 26.12a	101.60 ± 31.76a	125.13 ± 5.39a
34	3-Methyl-2-buten-1-ol	556–82–1	1315	1301	–	–	–	–	255.81 ± 30.09	252.07 ± 12.27
35	n-Hexanol	111–27-3	1348	1360	244.00 ± 28.36d	395.41 ± 71.91bc	489.84 ± 7.71ab	545.15 ± 155.66a	437.18 ± 48.12abc	344.04 ± 35.62 cd
36	1-Octen-3-ol	3391-86-4	1444	1430	150.17 ± 31.19	204.11 ± 29.46	194.39 ± 25.23	270.43 ± 48.82	–	–
37	2-Ethylhexanol	104–76–7	1484	1480	1464.28 ± 363.64ab	1400.98 ± 132.65b	1450.26 ± 151.95ab	1846.04 ± 111.99a	1189.90 ± 284.46bc	969.81 ± 125.81c
38	Linalool	78–70-6	1538	1554	74.20 ± 1.80c	115.84 ± 23.75c	157.17 ± 49.59c	252.75 ± 78.00b	380.55 ± 72.20a	453.96 ± 27.72a
39	1-Methyl-4-(1-methylethenyl) cyclohexanol	138–87-4	1621	1616	–	77.37 ± 5.04	26.70 ± 4.35	–	–	–
40	α,α-4-Trimethylcyclohexylmethanol	21,129–27-1	1632	NF	3265.33 ± 276.23a	2981.43 ± 286.95a	2945.56 ± 161.7a	2799.61 ± 432.70ab	2360.12 ± 37.68b	1721.64 ± 216.14c
41	1-Nonanol	143–08-8	1653	1640	929.44 ± 112.43bc	1220.53 ± 93.57a	1369.55 ± 291.85a	1107.46 ± 12.60ab	704.17 ± 195.19 cd	622.94 ± 59.21d
42	α-Terpineol	98–55-5	1687	1680	264.80 ± 7.3d	1023.41 ± 74.88b	1002.62 ± 142.65b	2023.94 ± 212.73a	1639.22 ± 582.85a	96.29 ± 13.03d
43	Geraniol	106–24-1	1841	1841	–	60.20 ± 6.98	192.60 ± 0.18	618.56 ± 102.42	763.95 ± 215.42	702.96 ± 58.61
44	Dodecanol	112–53-8	1960	1983	79.16 ± 13.08a	81.06 ± 23.12a	72.14 ± 27.24a	82.25 ± 30.05a	68.50 ± 82.44a	63.11 ± 1.35a
	Total alcohols				6590.42	7691.05	8033.97	9686.82	7901.00	5351.95

	Hydrocarbons (4)									
45	Dipentene	138–86-3	1191	1185	823.97 ± 103.83b	821.12 ± 155.67b	1003.54 ± 145.11b	2124.18 ± 266.43a	887.00 ± 192.27b	465.71 ± 47.84c
46	Styrene	100–42-5	1247	1254	25.50 ± 6.31c	56.79 ± 11.90c	238.35 ± 19.57ab	180.58 ± 33.44b	221.06 ± 63.00ab	258.16 ± 18.33a
47	Tetradecane	629–59-4	1390	1400	58.45 ± 46.18b	109.67 ± 19.05ab	81.23 ± 9.29ab	114.90 ± 19.45ab	147.20 ± 53.49a	75.40 ± 12.81ab
48	1,5-Dodecadiene	NF	1814	NF	133.22 ± 33.62	128.03 ± 20.62	87.12 ± 4.03	110.88 ± 32.13	–	–
	Total hydrocarbons				1041.14	1115.61	1410.24	2530.54	1255.26	799.27

	Others (9)									
49	Toluene	108–88-3	1032	1036	–	–	5.97 ± 6.16	–	–	19.63 ± 1.86
50	2-Pentylfuran	3777-69-3	1225	1249	159.62 ± 29.71a	156.79 ± 14.68a	192.81 ± 44a	152.82 ± 3.68a	209.74 ± 60.10a	230.08 ± 28.77a
51	2-Methylpyrazine	109–08-0	1257	1265	343.74 ± 48.84a	307.16 ± 29.68ab	237.79 ± 17.61b	375.89 ± 67.28a	304.67 ± 28.01ab	297.33 ± 84.31ab
52	2,5-Dimethylpyrazine	123–32-0	1313	1321	146.64 ± 17.03	123.91 ± 12.46	108.11 ± 18.32	129.35 ± 5.37	–	–
53	2,6-Dimethylpyrazine	108–50-9	1319	1330	57.19 ± 5.73	84.15 ± 13.29	44.90 ± 1.86	140.07 ± 19.62	–	–
54	Tea pyrrole	2167-14-8	1595	1616	85.42 ± 6.67d	148.57 ± 35.85bc	194.76 ± 40.94b	361.04 ± 1.29a	117.78 ± 4.98 cd	31.09 ± 5.38e
55	Dimethyl sulfone	67–71-0	1884	1912	25.93 ± 2.66	35.70 ± 18.61	30.66 ± 9.53	98.33 ± 52.61	35.97 ± 14.28	–
56	Butylhydroxytoluene	128–37-0	1900	1911	371.34 ± 59.2a	385.2 ± 50.95a	405.61 ± 9.37a	470.55 ± 99.23a	471.58 ± 417.22a	267.88 ± 43.95a
57	Toluene	118–71-8	1965	1965	–	–	–	17.02 ± 6.86	38.81 ± 51.15	–
	Total others				1189.88	1241.48	1214.64	1745.07	1178.55	826.38

Note: Results are presented as the mean ± standard deviation. Lowercase letters indicate significant differences within each column (*P* < 0.05).

RI (retention index) was calculated by analyzing a C7–C40 alkane mixture under the above GC–MS condition.

RI_ref_ (retention index reference) referred from web: https://webbook.nist.gov/chemistry/name-ser/.

—, means not detected.

The contributions of aroma-active compounds are not only determined by concentrations but also by threshold values. Based on the principle of OAVs, styrene, linalool, caprylic acid, and 1-nonanol were considered as contributors to the flavour of fermented milk (Fig. 2C). The OAV values of other volatiles were below 1, which meant that they would play a supplementary role in the overall flavour of fermented milk (Tian et al., 2019; H. Xu et al., 2024).

The results of the principal component analysis Fig. 2D show that before 12 h of fermentation time, the flavour of the samples changed less and it was almost difficult to separate them from the distribution distance, but after 24 h the volatiles changed significantly.

#### 3.2.2 Electronic nose

**Fig. 3:**
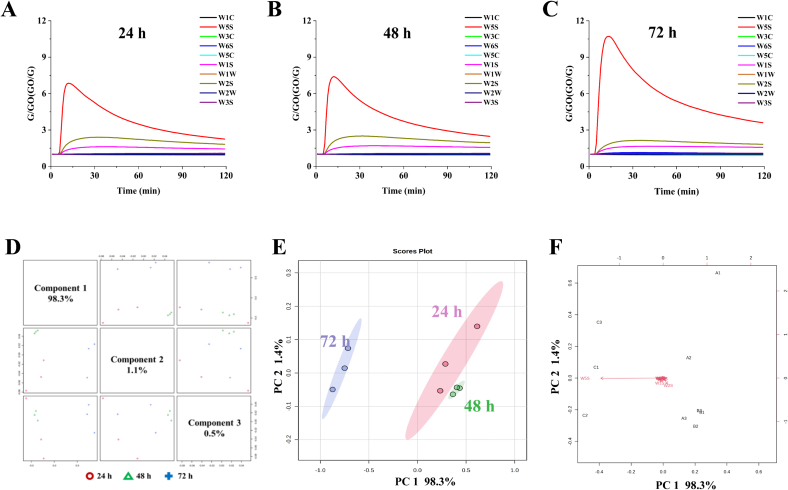
Response curve based on the results of electronic nose at fermented 24 h (A); Response curve based on the results of electronic nose at fermented 48 h (B); Response curve based on the results of electronic nose at fermented 72 h (C); The pairwise score plot for top 3 pcs of *E*-nose responses (D); The PCA scores plot of E-nose responses (E); The PCA biplot of E-nose responses (F).

Based on the results of the fermentation process, the fermentation characteristics of the yogurt and the type and concentration of volatiles, it was concluded that fermentation for at least 20 h was necessary to obtain a relatively stable product. Therefore, we analysed the fermented milk at three times, 24 h, 48 h and 72 h, by means of an electronic nose to obtain an overall odour profile. T The response curves of the electronic nose (Fig. 3A-C) showed that the overall response trend of the samples was consistent with the response characteristic of yogurt. However, at the initial stage of *E*-nose detection, the response profile was similar for the 24 h and 48 h samples, but the W5S response value was significantly higher for the 72 h sample. The data selected for stabilization at the end of the E-nose test phase is shown in the radar plot, where the W2W sensor has a response value below 1, which is less useful for distinguishing between samples with different fermentation times. Meanwhile, the other nine sensors contributed to varying degrees. The top 3 principal components explained the variance, showing a cumulative contribution of 99.9%. This result indicated that the main components were able to reflect all the information on volatiles of 3-time nodes during fermentation (Fig. 3D). Notably, the differences in clustering were observed in the PCA score plot for the 3-time nodes during fermentation (Fig. 3E). The spatial distribution and distances of aromas in fermented milk samples were further analysed using PCA, and the results are presented in Fig. 3F. The results demonstrated that viscosity and elasticity played a crucial role in the flavour release and retention during fermentation (Peng et al., 2023).

#### 3.2.3 Sensory evaluation

**Fig. 4:**
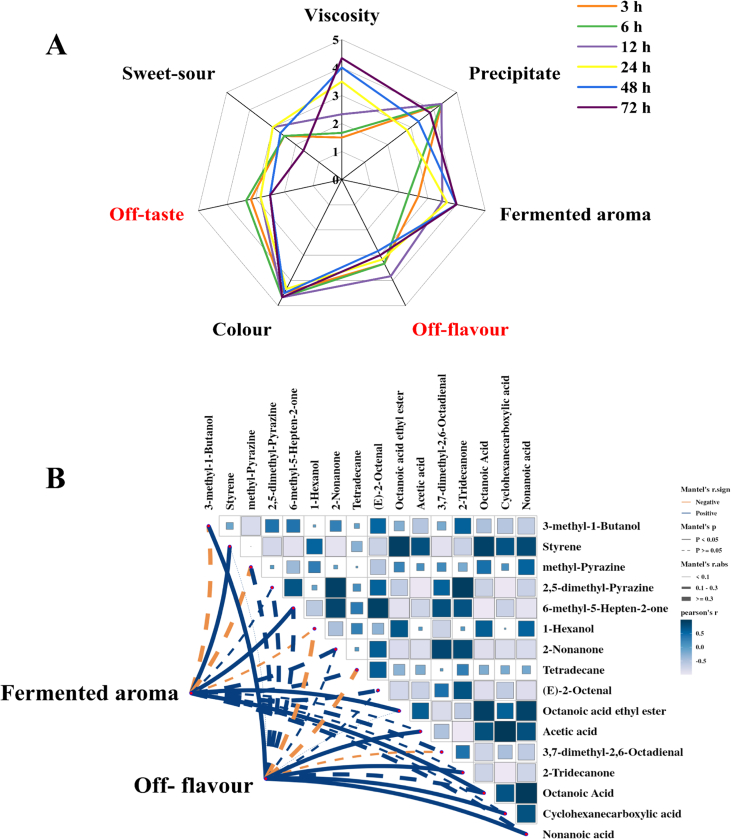
Rada chart based the results of sensory evaluation of fermented milk at different fermentation time (A); Correlation between sensory properties and volatile compounds (B).

During the later stages of fermentation, especially after 40 h of fermentation, the quality of the fermented milk reached a steady state, including rheological properties, volatiles, and odour profile. Therefore, fermented milks with 24 h, 48 h and 72 h of fermentation were selected for sensory evaluation. The evaluation rated seven sensory attributes obtained from the pre-screening, and these results were presented in the form of radar charts (Fig. 4).

The highest-rated fermented milk was at 72 h for all three indicators: viscosity, sedimentation status, and colour. The taste results showed that the fermented milk (24 h) had a moderate sour-sweet ratio and the sour-sweet flavour deteriorated and the off-taste became more obvious with the extension of fermentation time, but there was no difference between 48 h and 72 h.

The odour test consisting of fermentation aroma and off-flavour enhanced and reached at a peak after 72 h with the extension of fermentation time. The off-flavour was most pronounced at 48 h and then gradually weakened. In conclusion, fermented milk with 72 h of fermentation was the best, the fermented milk with 24 h of fermentation had the best flavour, fermented milk with 48 h of fermentation had the strongest aroma, and fermented milk with 24 h of fermentation had the weakest off-flavour.

The correlations between volatile compounds and sensory attributes were analysed. Fig. 4B shows the coefficients of the Pearson's correlation between volatiles and odour. Data demonstrated a significant positive correlation (*P* < 0.05) among fermented aroma and styrene (balsam, gasoline), 6-methyl-5-hepten-2-one (citrus, lemon), octanoic acid ethyl ester (fruity, sweet), octanoic acid (sweat, cheese) (*r* = 0.871; 0.691; 0.790, and 0.548, respectively) (Wang et al., 2024). Styrene acts as an odour-active substance and it contributes to fermentation aroma. In this study, 6-methyl-5-hepten-2-one provided a pronounced fruity flavour contributed to the overall sensory quality. Meanwhile, it served as the main source of linalool, one of the odour-active compounds. However, a significant positive correlation (*P* < 0.05) was observed among off-flavour, 3-methyl-1-butanol, acetic acid, 2-tridecanone, cyclohexanecarboxylic acid, nonanoic acid (*r* = 0.820; 0.427; 0.436; 0.430, and 0.489, respectively). 3-methyl-1-butanol has been described as a whiskey-like odour note, in general, awful smell (Wang et al., 2024). Furthermore, the concentration of 3-methyl-1-butanol is negatively correlated with the fermentation aroma of fermented milk. Nonanoic acid was reported as having a fatty characteristic odour and a corresponding unpleasant taste, which would cause a negative impact on the overall flavour (Ning et al., 2011). The acetic acid in high concentration may impart an unpleasant vinegar odour and lead to strong off-flavour.

### 3.3 Fermentation characteristics of fermented milk with prebiotics

**Fig. 5:**
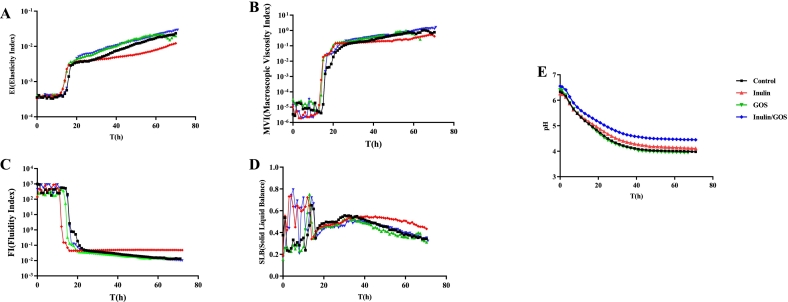
Microrheological properties and pH of fermented milk added prebiotics during fermentation. Elasticity index (A); Macroscopic Viscosity Index (B); Fluidity Index (C); Solid-Liquid Balance (D); pH (E). Legend: Black: Control; Red: Inulin; Class A; Green: GOS; Blue: Inulin/GOS. (For interpretation of the references to colour in this figure legend, the reader is referred to the web version of this article.)

As shown in Fig. 5, all fermented milk samples had the same pH, indicating that the prebiotics did not have an effect on the activity of L. *helveticus*, which was in agreement with previous publications (Wu, Dai, et al., 2023; K. Xu et al., 2019). The effect of added prebiotics on the rheological properties of fermented milk is shown in Fig. 5A-D. Prebiotics had a large effect on the rheological properties of fermented milk, in which the sample with added inulin had the lowest elasticity, solid-liquid equilibrium point, viscosity, and the strongest fluidity. The results indicated that it had the loosest organization after gelation and lower gel strength, while the rheological properties of the control, GOS and compounded samples were similar, and the fluidity of the three samples was almost the same.

In comparison, the sample fermented with inulin/GOS showed the highest elasticity and viscosity, and the sample with GOS showed the lowest SLB. The addition of inulin/GOS improved the rheological properties of the samples, while inulin decreased the viscoelasticity of the samples, which may be attributed to the fact that the L. *helveticus* could produce extracellular polysaccharides during fermentation to enhance the gel structure.

The pH change of fermented milk with different prebiotics were shown in Fig. 5E. There was no change in pH of fermented milk with the addition of GOS as compared to the control group. In contrast, the addition of inulin or inulin/GOS resulted in an increase in pH at the fermentation endpoint of fermented milks. But the effect was not significant.

### 3.4 The performance of flavour in fermented milk with prebiotics

#### 3.4.1 GC–MS

The samples at 24 h (Supplementary Table 2), 48 h (Supplementary Table 3), and 72 h (Supplementary Table 4) of fermentation were taken, and the concentrations of the volatiles were identified through HS-SPME/GC–MS to investigate the effect of prebiotics on the flavour release of fermented milk. Significant decreases in aldehyde concentrations were observed with prebiotics.

The GOS samples showed the greatest reduction in acetaldehyde concentration, while the reduction was smaller in inulin samples. Significant decreases in aldehyde were observed with prebiotics. The GOS samples showed the greatest reduction in acetaldehyde concentration, while the reduction was smaller in inulin samples. The concentration of diacetyl in fermented milk decreased after the addition of prebiotics, especially inulin/GOS samples. Pyruvate can be converted from glucose through the Embden-Meyerhof-Parnas Pathway in fermented milk, which is further converted into diacetyl, acetaldehyde, and other volatile flavour compounds (Sun et al., 2023). Acetoin is the reduced form of diacetyl, and its concentration increased significantly with prebiotics, probably because the addition of prebiotics hindered the synthesis of diacetyl or enhanced the synthesis of Acetoin. The concentration of most alcohols decreased to different degrees after the addition of prebiotics but increased the types of alcohols and enriched the flavour of fermented milk.

The most significant reduction in hydrocarbons concentration was observed in the samples with inulin/GOS. All key flavour substances of fermented milk showed a decrease in concentration with the addition of prebiotics. In fermented milk supplemented with inulin/GOS, the decreased concentration of all key flavour compounds was the most significant except linalool, probably because the synergistic effect enhanced the ability of the single prebiotics.

#### 3.4.2 Sensory evaluation

**Fig. 6:**
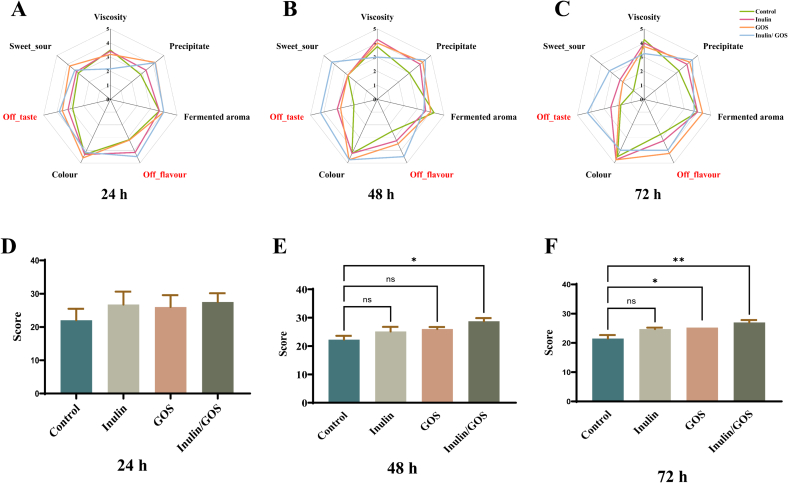
Sensory evaluation results of fermented milk with prebiotics. Rada chart of attributes at fermented 24 h (A), 48 h (B), 72 h (C); Histogram of gross scores at fermented 24 h (D), 48 h (E), and 72 h (F). Significant difference between two groups: “ns” indicates no significant difference, * *P* < 0.05, ** *P* < 0.01.

Fig. 6 demonstrated the results of sensory evaluation after the addition of prebiotics. The addition of prebiotics had a significant effect on the sensory scores, including the improvement of taste, aroma and tissue state. The gel strength of the samples was enhanced by the addition of prebiotics, which not only improved the tissue state, but also made it more difficult for the flavour to be dispersed out of the samples including fermented aroma and off-flavour (Fig. 6A-C).

There was no significant difference in the overall sensory quality between each group at 24 h. However, compared with the control group, the total sensory evaluation scores were all improved with the addition of prebiotics during fermentation (Fig. 6D). The results of volatile compounds suggested that the addition of prebiotics promoted the release of flavour substances in the matrix of fermented milk samples and enriched the flavour. Meanwhile, the overall texture of fermented milk became better and the elasticity was improved due to the reduced sedimentation. At 48 h of fermentation, a significant improvement in the inulin/GOS group was observed compared to the control group (Fig. 6E), in terms of texture, sweet-sour, and off-flavour. At 72 h of fermentation, the total score of GOS (*P* < 0.05) and inulin/GOS group was significantly (*P* < 0.01) higher than that of the control group (Fig. 6F). Compared with the control, the overall scores of the samples were higher after the addition of prebiotics, indicating that the addition of prebiotics significantly improved the quality of the samples.

## 4 Conclusions

This study aimed to track the variations of the volatile components and rheological properties in L. *helveticus* fermented milk throughout the fermentation process and explore prebiotic fermented milk from a sensory perspective. The quality of the fermented milk prepared using L. *helveticus* as a starter tended to be stable after 24 h, with the fermentation aroma reaching its highest at 72 h and the off-flavour being the weakest at 48 h. Styrene, Linalool, Octanoic acid, and 1-Nonanol were the key flavour active substances during fermentation. The type of prebiotics affected some physicochemical and sensory properties of the fermented milk. Compared with single inulin prebiotics and GOS prebiotics, the blend addition of inulin/GOS probiotics significantly improved the texture and flavour release of fermented milk. To some extent, these results provide new insight into the dynamic changes of fermented milk during fermentation, further investigation of the Starter and prebiotics conditions on the quality and flavour of processed dairy products is warranted.

## CRediT authorship contribution statement

**Xuelu Chi:** Writing – original draft, Methodology, Investigation, Conceptualization. **Qingyu Yang:** Writing – review & editing. **Yufang Su:** Writing – review & editing, Conceptualization. **Jian Zhang:** Writing – review & editing. **Baoguo Sun:** Writing – review & editing, Conceptualization. **Nasi Ai:** Writing – review & editing, Project administration, Funding acquisition, Conceptualization.

## Declaration of competing interest

The authors declare that they have no known competing financial interests or personal relationships that could have appeared to influence the work reported in this paper.

## Data Availability

Data will be made available on request.

## Appendix A Supplementary data

Supplementary material Image 1
