# Supplementary material for: Improvement of rheological and sensory properties of Lactobacillus helveticus fermented milk by prebiotics
Source: Food Chem X. 2024 Jul 20;23:101679. doi: 10.1016/j.fochx.2024.101679 (PMC11338119; doi:10.1016/j.fochx.2024.101679)
Supplement: Supplementary file 1 — Supplementary material [file mmc1.docx]

**Supplementary data**

Supplementary Table 1 Sensory evaluation criteria.

Supplementary Table 2 Quantitative results of VOCs in fermented milk at 24 h.

Supplementary Table 3 Quantitative results of VOCs in fermented milk at 48 h.

Supplementary Table 4 Quantitative results of VOCs in fermented milk at 72 h.

Supplementary Figure 1 Total ion flow diagram for fermented milk.

Supplementary Table 1 Sensory evaluation criteria

| Items | | Attributes | Scores |
| --- | --- | --- | --- |
| visual | viscosity | Higher viscosity | 4~5 |
|  |  | Lower viscosity | 2~3 |
|  |  | Dilute solutions | 0~1 |
|  | texture | No precipitation | 4~5 |
|  |  | A little precipitation | 2~3 |
|  |  | A large amount of precipitation | 0~1 |
|  | colour | Milky white, uniformly | 4~5 |
|  |  | Light yellow, uniformly | 2~3 |
|  |  | Yellow, non-uniformly | 0~1 |
| Odour | Fermented aroma | Intense fermented aroma | 4~5 |
|  |  | Light fermented aroma | 2~3 |
|  |  | Almost no smell | 0~1 |
|  | Off-flavour | Almost no smell | 4~5 |
|  |  | Light off-flavour | 2~3 |
|  |  | Intense off-flavour | 0~1 |
| Taste | Off-taste | Almost no off-taste | 4~5 |
|  |  | Light off-taste | 2~3 |
|  |  | Intense off-taste | 0~1 |
|  | Sour-sweet | Excellent sour and sweet taste | 4~5 |
|  |  | Moderate sour and sweet taste | 2~3 |
|  |  | Inappropriate sour and sweet taste | 0~1 |

Supplementary Table 2 Quantitative results of VOCs in fermented milk at 24 h

| No. | compounds | Concentration (μg/L) | | | |
| --- | --- | --- | --- | --- | --- |
|  |  | Control | Inulin | GOS | Inulin/GOS |
|  | Aldehydes (7) |  |  |  |  |
| 1 | Acetaldehyde | 1782.52±105.67 | 601.41±37.51 | 110.86±23.32 | 157.74±20.81 |
| 2 | Nonanal | 412.63±68.48 | — | — | — |
| 3 | Nonyl aldehyde | — | 443.50±140.75 | 99.57±9.50 | 29.14±3.03 |
| 4 | 2-Undecylenal | — | — | — | 3.94±0.57 |
| 5 | Trans-2-dodecenal | — | — | 8.74±2.79 | — |
| 6 | Benzaldehyde | — | 45.38±11.74 | 20.15±0.94 | — |
| 7 | Citral | — | — | — | 6.93±1.72 |
|  | Ketones (14) |  |  |  |  |
| 8 | 2-Heptanone | — | 365.18±52.58 | 349.26±96.46 | 5.50±0.24 |
| 9 | Acetyl methyl carbinol | 134.12±31.84 | — | — | — |
| 10 | Acetoin | — | 156.02±23.03 | 142.18±18.72 | 70.25±21.83 |
| 11 | Hydroxyacetone | — | — | 10.77±0.86 | 22.82±11.81 |
| 12 | Methyl heptenone | — | 60.82±1.61 | 4.59±0.47 | 1.74±0.27 |
| 13 | 2-Nonanone | 609.14±23.94 | 470.14±97.98 | 437.18±83.47 | 239.51±48.63 |
| 14 | Menthone | — | 25.85±2.92 | 395.96±10.49 | 29.02±6.47 |
| 15 | 3,5-Octandien-2-one | — | — | 12.09±3.14 | 2.96±1.66 |
| 16 | 2-Undecone | — | 480.24±55.39 | 420.08±88.26 | 128.19±20.84 |
| 17 | 2-Tridecone | — | 19.37±0.58 | 63.69±9.75 | 6.03±1.04 |
| 18 | 2-Undecanone | 1139.48±41.23 | — | — | — |
| 19 | 1,6-Dihydrocarvone | 14.81±4.89 | — | — | — |
| 20 | 2-Tridecanone | 194.07±46.42 | — | — | — |
| 21 | 2-Pentadecanone | 125.30±24.20 | — | 29.30±4.21 | 18.41±0.85 |
|  | Esters (5) |  |  |  |  |
| 22 | Isobutyl butyrate | — | 38.31±5.06 | 42.39±6.93 | 3.22±0.60 |
| 23 | Ethyl caprylate | 60.56±2.4 | — | — | — |
| 24 | Amyl pelargonate | 230.81±6.86 | — | — | — |
| 25 | Butyl butyrate | — | 220.72±31.15 | — | 29.26±2.05 |
| 26 | Methyl Cyclopropane carboxylate | — | — | — | 8.27±0.51 |
|  | Acids (11) |  |  |  |  |
| 27 | Acetic acid | 763.58±96.60 | 4.64±1.33 | 85.24±17.13 | 0.44±0.18 |
| 28 | Caproic acid | 2953.57±251.56 | — | — | — |
| 29 | Octanoic acid | 5552.94±607.39 | — | — | — |
| 30 | Butyric acid | — | — | 174.85±2.11 | — |
| 31 | Hexanoic acid | — | 1127.74±205.50 | 359.20±30.02 | 498.74±22.71 |
| 32 | Heptanoic acid | — | 44.96±6.58 | 18.75±1.52 | — |
| 33 | Caprylic acid | — | 1280.14±53.64 | 966.48±100.02 | 398.12±68.21 |
| 34 | Nonanoic acid | 229.32±3.82 | 29.50±5.38 | 42.67±2.25 | 14.76±6.71 |
| 35 | Decanoic acid | 5131.99±921.25 | 480.05±155.09 | 585.07±106.72 | 294.25±77.46 |
| 36 | 9-Decenoic acid | 742.58±32.67 | 133.23±37.53 | 288.98±63.59 | 144.53±27.26 |
| 37 | Lauric acid | 1884.23±245.88 | 188.37±20.99 | 213.94±26.92 | 109.38±25.01 |
|  | Alcohols (23) |  |  |  |  |
| 38 | Isopentenol | 140.63±26.12 | — | — | — |
| 39 | 3-Methyl-1-butanol | — | 143.03±30.25 | 128.89±4.44 | 90.79±17.62 |
| 40 | trans-2-penten-1-ol | — | 35.11±6.20 | — | 27.98±0.89 |
| 41 | 3-Methyl-2-buten-1-ol | — | 124.17±27.60 | — | 79.43±15.25 |
| 42 | 1-Hexanol | 545.15±155.66 | 325.45±57.04 | 282.15±22.03 | 150.98±26.89 |
| 43 | 2-Methylhexadecan-1-ol | — | 3.37±0.02 | — | — |
| 44 | 1-Octen-3-ol | 270.43±48.82 | 64.69±10.28 | 75.51±11.19 | 27.44±3.45 |
| 45 | 2-Ethylhexanol | 1846.04±111.99 | 487.15±24.04 | 280.25±60.38 | 199.16±34.36 |
| 46 | 2-Nonanol | — | 12.01±3.66 | 9.18±0.60 | 2.05±0.52 |
| 47 | Linalool | 252.75±78.00 | 125.89±28.87 | 33.94±8.03 | 15.23±1.40 |
| 48 | α,α-4-Trimethylcyclohexylmethanol | 2799.61±432.70 | — | — | — |
| 49 | 1-Octanol | — | 320.98±83.65 | 532.32±61.81 | 229.25±32.08 |
| 50 | (+)-Isopulegol | — | — | — | 1.28±0.01 |
| 51 | Dihydro-α-terpineol | — | 850.66±56.05 | 974.49±190.50 | 320.13±66.68 |
| 52 | 1-Nonanol | 1107.46±12.60 | 309.91±67.05 | 402.21±62.40 | 135.12±29.24 |
| 53 | Alpha-Terpineol | 2023.94±212.73 | 37.86±1.19 | 4.85±1.56 | 15.56±4.88 |
| 54 | Geraniol | 618.56±102.42 | — | — | — |
| 55 | borneol | — | 12.20±1.22 | — | — |
| 56 | 2-Undecanol | — | 17.06±2.35 | 8.16±0.05 | 6.34±2.38 |
| 57 | Myrtenol | — | 60.06±10.01 | — | — |
| 58 | 2,7-Dimethyl-2,6-octadiene | — | 178.02±1.93 | 146.74±33.80 | 63.33±3.59 |
| 59 | 2-Tridecanol | — | — | 13.93±1.43 | — |
| 60 | 1-Dodecanol | 82.25±30.05 | 7.03±1.34 | 53.88±16.29 | 2.66±0.16 |
|  | Hydrocarbons (7) |  |  |  |  |
| 61 | Undecane | — | — | — | 33.79±4.97 |
| 62 | Dipentene | 2124.18±266.43 | — | — | — |
| 63 | Limonene | — | 548.17±81.63 | 125.61±3.88 | — |
| 64 | Styrene | 180.58±33.44 | — | — | 6.85±0.05 |
| 65 | Tridecane | — | 154.94±45.00 | 268.16±9.65 | — |
| 66 | Tetradecane | 114.90±19.45 | 87.90±2.56 | 37.60±9.65 | — |
| 67 | 1,5-Dodecadiene | 110.88±32.13 | — | — | 3.61±0.25 |
|  | Others (12) |  |  |  |  |
| 68 | 2-Pentylfuran | 152.82±3.68 | 75.73±9.17 | 38.60±1.82 | 16.70±3.18 |
| 69 | 2-Methylpyrazine | 375.89±67.28 | 176.30±37.67 | 221.46±25.97 | 96.88±21.11 |
| 70 | 2,5-Dimethylpyrazine | 129.35±5.37 | — | 135.80±33.94 | — |
| 71 | 2,6-Dimethylpyrazine | 140.07±19.62 | 43.21±2.63 | 126.32±13.60 | 58.83±10.57 |
| 72 | 4-Methyl-2-(2-methyl-1-propenyl)tetrahydropyran | — | — | — | — |
| 73 | Tea pyrrole | 361.04±1.29 | — | — | — |
| 74 | Trimethyl-pyrazine | — | — | 4.32±0.13 | — |
| 75 | 1-Ethyl-1H-pyrrole-2-carbaldehyde | — | 18.41±1.83 | 19.86±5.84 | 4.85±1.54 |
| 76 | Dimethyl sulfone | 98.33±52.61 | 22.33±1.22 | 16.67±0.13 | 16.41±1.93 |
| 77 | Butylated Hydroxytoluene | — | 404.07±91.28 | 1167.33±111.05 | 277.98±52.59 |
| 78 | Butylhydroxytoluene | 470.55±99.23 | — | — | — |
| 79 | Toluene | 17.02±6.86 | — | — | — |

—, means not detected.

Supplementary Table 3 Quantitative results of VOCs in fermented milk at 48 h

| No. | compounds | Concentration(μg/L) | | | |
| --- | --- | --- | --- | --- | --- |
|  |  | Control | Inulin | GOS | Inulin/GOS |
|  | Aldehydes (5) |  |  |  |  |
| 1 | Acetaldehyde | 1519.5±122.57 | 2928.50±124.89 | — | 348.75±25.61 |
| 2 | Nonanal | 541.50±88.74 | — | — | — |
| 3 | Nonyl aldehyde | — | 131.12±27.27 | 70.19±7.86 | 89.70±18.68 |
| 4 | Benzaldehyde | 123.68±18.80 | 16.74±1.57 | 18.60±6.10 | 15.18±2.54 |
| 5 | Citral | — | — | — | 1.63±0.42 |
|  | Ketones (13) |  |  |  |  |
| 6 | 2-Heptanone | 494.72±65.76 | 237.09±10.75 | 497.68±83.39 | 362.28±59.06 |
| 7 | Acetyl methyl carbinol | 234.02±65.60 | — | — | — |
| 8 | Acetoin | — | 397.48±46.80 | 303.43±61.72 | 362.22±47.47 |
| 9 | Hydroxyacetone | — | 18.05±2.66 | 16.06±3.87 | 38.42±13.14 |
| 10 | Methyl heptenone | — | 10.95±3.22 | — | 3.36±0.72 |
| 11 | 2-Nonanone | 479.26±99.12 | 225.16±22.76 | 433.76±94.30 | 325.25±83.66 |
| 12 | Menthone | — | 14.75±2.56 | 84.14±16.18 | 47.05±2.93 |
| 13 | 3,5-Octandien-2-one | — | — | 7.77±2.28 | — |
| 14 | 2-Undecone | — | 162.91±29.18 | 346.35±66.78 | 257.32±51.02 |
| 15 | 2-Tridecone | — | 12.63±0.66 | 35.95±3.75 | 10.06±1.22 |
| 16 | 2-Undecanone | 615.12±187.79 | — | — | — |
| 17 | 2-Tridecanone | 47.21±28.21 | — | — | — |
| 18 | 2-Pentadecanone | 53.93±81.39 | — | 20.90±2.67 | — |
|  | Esters (6) |  |  |  |  |
| 19 | Isobutyl butyrate | — | 21.18±2.79 | 2.96±0.93 | — |
| 20 | Ethyl caprylate | 60.74±17.13 | — | — | — |
| 21 | γ-Decalactone | 16.74±6.24 | — | — | — |
| 22 | Butyl butyrate | — | 220.92±3.70 | — | 91.82±18.39 |
| 23 | Methyl Cyclopropane carboxylate | — | — | — | 141.02±15.64 |
| 24 | 5-Decanolide | — | — | — | 2.33±0.10 |
|  | Acids (13) |  |  |  |  |
| 25 | Acetic acid | 4642.57±398.55 | 372.12±50.53 | 1445.83±146.76 | 3473.38±481.56 |
| 26 | Caproic acid | 6742.87±1134.67 | — | — | — |
| 27 | Octanoic acid | 6770.52±1544.76 | — | — | — |
| 28 | Butyric acid | — | — | 193.74±17.20 | — |
| 29 | Cyclohexanecarboxylic acid | 167.13±25.09 |  |  |  |
| 30 | Hexanoic acid | — | 1223.43±294.05 | 1078.17±63.24 | 1074.20±151.99 |
| 31 | Heptanoic acid | — | 25.17±5.00 | 52.21±9.71 | — |
| 32 | Caprylic acid | — | 1156.18±58.4 | 1112.15±146.73 | 1302.33±274.22 |
| 33 | Cyclohexanic acid | — | 18.94±3.66 | — | 41.90±3.96 |
| 34 | Nonanoic acid | 165.33±20.50 | 20.03±9.99 | — | 31.73±5.16 |
| 35 | Decanoic acid | 2365.37±347.15 | 380.26±128.88 | 640.14±171.02 | 608.97±60.98 |
| 36 | 9-Decenoic acid | 702.02±86.16 | 76.72±0.39 | 123.90±24.71 | 125.71±7.09 |
| 37 | Lauric acid | 817.57±167.98 | 46.84±11.73 | 150.54±31.98 | 99.77±15.89 |
|  | Alcohols (21) |  |  |  |  |
| 38 | Isopentenol | 101.60±31.76 | — | — | — |
| 39 | 3-Methyl-1-butanol | — | 106.84±4.23 | 116.50±2.37 | 79.35±4.95 |
| 40 | trans-2-penten-1-ol | — | 27.78±2.68 | — | — |
| 41 | 3-Methyl-2-buten-1-ol | 255.81±30.09 | 175.25±9.41 | 226.33±10.71 | 187.14±48.67 |
| 42 | 1-Hexanol | 437.18±48.12 | 211.68±11.95 | 283.80±44.01 | 279.46±19.55 |
| 43 | 1-Octen-3-ol | — | — | 90.75±8.22 | — |
| 44 | 2-Ethylhexanol | 1189.90±284.46 | 192.45±22.63 | 229.35±44.13 | 345.11±69.16 |
| 45 | 2-Nonanol | — | 7.17±2.34 | 17.73±2.04 | 16.54±2.62 |
| 46 | Linalool | 380.55±72.20 | 86.93±10.24 | 70.13±9.20 | 100.36±24.64 |
| 47 | α,α-4-Trimethylcyclohexylmethanol | 2360.12±37.68 | — | — | — |
| 48 | 1-Octanol | — | 149.76±17.10 | 409.47±78.18 | 280.43±38.59 |
| 49 | (+)-Isopulegol | — | — | — | 4.00±1.13 |
| 50 | Dihydro-α-terpineol | — | 456.56±55.44 | 841.18±125.16 | 1103.20±249.5 |
| 51 | 1-Nonanol | 704.17±195.19 | 146.78±26.96 | 268.15±29.45 | 199.50±16.60 |
| 52 | Alpha-Terpineol | 1639.22±582.85 | 63.87±7.23 | — | 68.48±11.66 |
| 53 | Geraniol | 763.95±215.42 | — | — | — |
| 54 | 2-Undecanol | — | 6.90±0.71 | 14.84±3.66 | 18.20±1.39 |
| 55 | Myrtenol | — | 30.05±5.28 | — | — |
| 56 | 2,7-Dimethyl-2,6-octadiene | — | 121.57±22.66 | 222.15±26.42 | 126.90±16.29 |
| 57 | 2-Tridecanol | — | — | 36.19±4.69 | — |
| 58 | 1-Dodecanol | 68.50±82.44 | 8.14±0.57 | 23.16±3.14 | 8.38±2.07 |
|  | Hydrocarbons (7) |  |  |  |  |
| 59 | Dipentene | 887.00±192.27 | — | — | — |
| 60 | Limonene | — | 77.91±16.27 | 209.92±8.42 | — |
| 61 | Styrene | 221.06±63.00 | — | — | 99.30±16.40 |
| 62 | Tridecane | — | — | 19.54±5.29 | — |
| 63 | Tetradecane | 147.20±53.49 | 8.45±0.70 | 10.90±0.61 | 100.03±13.61 |
| 64 | Cedrene | — | — | — | 8.64±0.75 |
| 65 | 1,5-Dodecadiene | — | — | — | 9.61±2.43 |
|  | Others (11) |  |  |  |  |
| 66 | Toluene | — | — | 10.54±1.44 | — |
| 67 | 2-Pentylfuran | 209.74±60.10 | 31.86±8.18 | 49.72±3.14 | 35.82±9.50 |
| 68 | 2-Methylpyrazine | 304.67±28.01 | 122.83±6.84 | 199.35±28.26 | 194.72±44.17 |
| 69 | 2,6-Dimethylpyrazine | — | — | 88.50±12.34 | 63.19±9.82 |
| 70 | 4-Methyl-2-(2-methyl-1-propenyl)tetrahydropyran | — | 1.61±0.65 | — | — |
| 71 | Tea pyrrole | 117.78±4.98cd | — | — | — |
| 72 | 1-Ethyl-1H-pyrrole-2-carbaldehyde | — | 8.71±2.49 | 15.40±0.22 | 8.77±1.01 |
| 73 | Dimethyl sulfone | 35.97±14.28 | 2.55±0.59 | — | 16.45±1.45 |
| 74 | Butylated Hydroxytoluene | — | 118.46±17.33 | 607.98±10.01 | 396.44±39.46 |
| 75 | Butylhydroxytoluene | 471.58±417.22 | — | — | — |
| 76 | Toluene | 38.81±51.15 | — | — | — |

—, means not detected.

Supplementary Table 3 Quantitative results of VOCs in fermented milk at 48 h

| No. | compounds | Concentration (μg/L) | | | |
| --- | --- | --- | --- | --- | --- |
|  |  | Control | Inulin | GOS | Inulin/GOS |
|  | Aldehydes (4) |  |  |  |  |
| 1 | Acetaldehyde | 14242.28±1724.07 | 263.99±1.86 | — | 1209.09±136.62 |
| 2 | Nonanal | 311.13±74.00 | — | — | — |
| 3 | Nonyl aldehyde | — | 314.65±96.81 | 82.35±46.02 | 186.39±45.69 |
| 4 | Benzaldehyde | 121.03±9.35 | 34.64±3.84 | 12.82±1.02 | 22.03±4.81 |
|  | Ketones (15) |  |  |  |  |
| 5 | Butane-2,3-dione | 382.40±80.13 | 167.56±22.16 | 0.68±0.14 | — |
| 6 | 2-Heptanone | 335.89±53.58 | 251.64±30.37 | 383.20±42.61 | 327.73±65.86 |
| 7 | Acetyl methyl carbinol | 628.33±100.11 | — | — | — |
| 8 | Acetoin | — | 2123.95±170.22 | 832.35±78.16 | 1294.25±146.08 |
| 9 | Hydroxyacetone | — | 30.62±4.44 | 27.36±4.85 | 52.89±8.98 |
| 10 | Methyl heptenone | — | 5.82±0.33 | — | 19.16±5.90 |
| 11 | 2-Nonanone | 462.34±43.36 | 243.04±25.57 | 350.58±20.04 | 312.73±55.48 |
| 12 | Menthone | — | 11.49±1.80 | 84.71±21.37 | 49.45±9.45 |
| 13 | 3,5-Octandien-2-one | — | 6.79±1.07 | 10.94±1.07 | — |
| 14 | 2-Undecone | — | 213.78±32.07 | 274.46±25.99 | 282.81±60.38 |
| 15 | 3-Tridecone | — | — | — | 114.99±3.00 |
| 16 | 2-Tridecone | — | 23.29±1.46 | 31.13±4.44 | 43.26±0.75 |
| 17 | 2-Undecanone | 577.71±78.30 | — | — | — |
| 18 | 2-Tridecanone | 53.57±10.24 | — | — | — |
| 19 | 2-Pentadecanone | 11.79±0.12 | — | 44.18±14 | — |
|  | Esters (5) |  |  |  |  |
| 20 | Isobutyl butyrate | — | 1.56±0.02 | 16.29±1.81 | — |
| 21 | Ethyl caprylate | 44.44±6.63 | — | — | — |
| 22 | γ-Decalactone | 10.09±0.82 | — | — | — |
| 23 | Butyl butyrate | — | 118.32±29.44 | — | — |
| 24 | Methyl Cyclopropane carboxylate | — | — | — | 158.16±26.83 |
|  | Acids (15) |  |  |  |  |
| 25 | Acetic acid | 5558.47±408.61 | 1513.47±309.57 | 5177.78±1012.61 | 2802.81±154.37 |
| 26 | Caproic acid | 9915.47±1418.45 | — | — | — |
| 27 | Octanoic acid | 5325.11±322.26 | — | — | — |
| 28 | Isovaleric acid | — | — | — | 50.50±9.78 |
| 29 | Butyric acid | — | — | 491.45±85.96 | 3964.53±246.9 |
| 30 | Cyclohexanecarboxylic acid | 148.98±8.05 | — | — | — |
| 31 | 2-Methylcaproic acid | — | — | — | 39.16±1.86 |
| 32 | Hexanoic acid | — | 1298.70±204.72 | 743.62±85.23 | 1495.16±69.85 |
| 33 | Heptanoic acid | — | 51.26±4.53 | 43.19±1.60 | — |
| 34 | Caprylic acid | — | 1411.01±340.32 | 1740±360.95 | 1827.05±119.03 |
| 35 | Cyclohexanic acid | — | 37.93±4.87 | 46.73±25.66 | 75.65±0.68 |
| 36 | Nonanoic acid | 193.72±30.14 | 25.44±3.79 | — | 70.64±1.10 |
| 37 | Decanoic acid | 3417.87±683.97 | 978.14±126.73 | 663.94±80.86 | 692.44±103.29 |
| 38 | 9-Decenoic acid | 383.29±6.37 | 145.88±36.06 | 283.26±72.02 | 179.87±35.12 |
| 39 | Lauric acid | 715.97±177.3 | 259.41±25.97 | 388.07±114.11 | 123.67±10.22 |
|  | Alcohols (20) |  |  |  |  |
| 40 | Isopentenol | 125.13±5.39 | — | — | — |
| 41 | 3-Methyl-1-butanol | — | 141.57±13.14 | 107.05±19.17 | 111.42±8.86 |
| 42 | trans-2-penten-1-ol | — | 35.72±5.37 | 100.40±16.27 | — |
| 43 | 3-Methyl-2-buten-1-ol | 252.07±12.27 | 240.42±15.81 | 310.80±68.04 | 179.10±21.43 |
| 44 | 1-Hexanol | 344.04±35.62 | 266.26±17.85 | 293.90±40.37 | 284.23±39.82 |
| 45 | 2-Ethylhexanol | 969.81±125.81 | 305.73±67.75 | 270.68±55.77 | 468.25±80.10 |
| 46 | 2-Nonanol | — | 14.67±4.25 | 27.38±1.21 | 24.73±4.51 |
| 47 | Linalool | 453.96±27.72 | 148.71±17.64 | 119.16±17.64 | 165.12±26.20 |
| 48 | α,α-4-Trimethylcyclohexylmethanol | 1721.64±216.14 | — | — | — |
| 49 | 1-Octanol | — | 218.40±25.85 | 363.57±48.89 | 369.65±63.69 |
| 50 | (+)-Isopulegol | — | — | — | 90.54±7.53 |
| 51 | Dihydro-α-terpineol | — | 601.27±56.29 | 2064.30±288.11 | — |
| 52 | 1-Nonanol | 622.94±59.21 | 338.06±53.15 | 289.60±59.33 | 301.43±38.48 |
| 53 | Alpha-Terpineol | 96.29±13.03 | 69.81±9.14 | — | 61.52±10.73 |
| 54 | Geraniol | 702.96±58.61 | — | — | — |
| 55 | 2-Undecanol | — | 8.46±2.10 | 31.65±0.32 | 22.74±0.68 |
| 56 | Myrtenol | — | 41.89±7.01 | — | — |
| 57 | 2,7-Dimethyl-2,6-octadiene | — | 208.26±37.67 | 226.67±1.04 | 168.16±32.64 |
| 58 | 2-Tridecanol | — | — | 19.06±6.54 | — |
| 59 | 1-Dodecanol | 63.11±1.35 | 12.01±2.24 | 27.72±8.08 | 7.18±0.01 |
|  | Hydrocarbons (7) |  |  |  |  |
| 60 | Dipentene | 465.71±47.84 | — | — | — |
| 61 | Limonene | — | 106.51±8.41 | 10.07±4.27 | — |
| 62 | Styrene | 258.16±18.33 | — | — | 117.27±21.57 |
| 63 | Tridecane | — | — | 8.02±2.61 | — |
| 64 | Tetradecane | 75.40±12.81 | 1.03±0.10 | 19.22±7.68 | 33.76±5.18 |
| 65 | Cedrene | — | — | — | 13.75±0.37 |
| 66 | 1,5-Dodecadiene | — | — | — | 25.15±4.32 |
|  | Others (10) |  |  |  |  |
| 67 | Toluene | 19.63±1.86 | 0.51±0.04 | — | — |
| 68 | 2-Pentylfuran | 230.08±28.77 | 44.91±11.56 | 6.01±2.26 | 20.19±2.20 |
| 69 | 2-Methylpyrazine | 297.33±84.31 | 152.43±22.42 | 200.06±30.77 | 182.79±35.26 |
| 70 | 2,6-Dimethylpyrazine | — | — | — | 47.75±6.58 |
| 71 | 4-Methyl-2-(2-methyl-1-propenyl)tetrahydropyran | — | 8.84±1.48 | — | — |
| 72 | Tea pyrrole | 31.09±5.38e | — | — | — |
| 73 | 1-Ethyl-1H-pyrrole-2-carbaldehyde | — | 6.17±1.31 | 24.45±5.25 | 13.78±0.16 |
| 74 | Dimethyl sulfone | — | 6.82±1.40 | — | 25.87±0.82 |
| 75 | Butylated Hydroxytoluene | — | 189.72±42.89 | 364.93±71.31 | 452.66±57.76 |
| 76 | Butylhydroxytoluene | 267.88±43.95 | — | — | — |

—, means not detected.

Supplementary Figure 1 Total ion flow diagram for fermented milk
